# Strong interactions between stoichiometric constraints and algal defenses: evidence from population dynamics of *Daphnia* and algae in phosphorus-limited microcosms

**DOI:** 10.1007/s00442-012-2404-y

**Published:** 2012-07-17

**Authors:** William R. DeMott, Ellen Van Donk

**Affiliations:** 1 Netherlands Institute of Ecology (NIOO-KNAW), Droevendaalsesteeg 10, 6708 PB Wageningen, The Netherlands; 2 Present Address: Department of Biology, Indiana–Purdue University, Fort Wayne, IN USA

**Keywords:** Food quality, Digestion-resistant algae, C:P ratio, Defense tradeoffs, TER models

## Abstract

The dynamic interactions among nutrients, algae and grazers were tested in a 2 × 3 factorial microcosm experiment that manipulated grazers (*Daphnia* present or absent) and algal composition (single species cultures and mixtures of an undefended and a digestion-resistant green alga). The experiment was run for 25 days in 10-L carboys under mesotrophic conditions that quickly led to strong phosphorus limitation of algal growth (TP ≅ 0.5 μM, N:P 40:1). Four-day *Daphnia* juvenile growth assays tested for *Daphnia* P-limitation and nutrient-dependent or grazer-induced algal defenses. The maximal algal growth rate of undefended *Ankistrodesmus* (mean ± SE for three replicate microcosms; 0.92 ± 0.02 day^−1^) was higher than for defended *Oocystis* (0.62 ± 0.03 day^−1^), but by day 6, algal growth was strongly P-limited in all six treatments (molar C:P ratio >900). The P-deficient algae were poor quality resources in all three algal treatments. However, *Daphnia* population growth, reproduction, and survival were much lower in the digestion-resistant treatment even though growth assays provided evidence for *Daphnia* P-limitation in only the undefended and mixed treatments. Growth assays provided little or no support for simple threshold element ratio (TER) models that fail to consider algae defenses that result in viable gut passage. Our results show that strong P-limitation of algal growth enhances the defenses of a digestion-resistant alga, favoring high abundance of well-defended algae and energy limitation of zooplankton growth.

## Introduction

Stoichiometric constraints can reduce food quality for many aquatic and terrestrial herbivores (Elser et al. 2000; Sterner and Elser 2002). *Daphnia* and phosphorus have received much attention in part because of their importance in lakes, and in part because daphniids have high requirements for P compared to other crustacean zooplankton. Threshold element ratio (TER) models predict the resource C:nutrient (usually N or P) ratio at which the grazer’s growth shifts from energy to nutrient limitation, taking into account the grazer’s nutrient content, ingestion rate, assimilation efficiencies (AEs), respiration costs, and nutrient excretion. The predicted molar C:P TER for *Daphnia* ranges from about 200 at high food to 230 at low food concentration (Anderson and Hessen 2005). TER models have been developed using laboratory data for high quality, undefended green algae. Thus, TER models include food quantity but, thus far, have not accounted for algal defenses that reduce ingestion rate or AE.

Deficiencies in essential fatty acids (EFAs) and algal digestion defenses have been proposed as alternatives to *Daphnia* P-limitation (reviewed by Sterner and Schulz 1998; Moe et al. 2005). Evidence that algae grown under nutrient limitation produce less EFAs supported an hypothesized interaction between P limitation and EFA limitation in *Daphnia* (Müller-Navarra 1995). However, while essential fatty acids are important for zooplankton growth (reviewed in Ravet et al. 2010), EFA supplements do not stimulate the growth of *Daphnia* feeding on strongly P-deficient green algae (Boersma 2000; Plath and Boersma 2001), while supplements of phosphate and phosphorus-rich but EFA-deficient cyanobacteria do (DeMott 1998).

Certain green algae that are high quality, undefended resources when grown without nutrient limitation develop thicker cell walls that resist digestion when grown under P-limitation (Van Donk and Hessen 1993; Van Donk et al. 1997). Since an increased proportion of cells survive gut passage, digestion resistance acts as a defense. We describe such algae as having “nutrient-dependent defenses” to distinguish them from constitutive defenses that are expressed in nutrient sufficient cultures (reviewed by Sterner 1989) and defenses that are induced by a grazer’s chemical or physical presence (reviewed by Van Donk et al. 2011). The terrestrial plant literature provides many examples in which constitutive defenses against herbivores are strengthened following leaf damage or are modulated by drought, nutrient limitation, or other stressors (reviewed by Stamp 2003). Which algae strengthen their defenses when nutrients are limiting and how strongly do these nutrient-dependent defenses vary between algal species? As far as we know, no study has tested whether planktonic algae with constitutive digestion defenses under nutrient-sufficient conditions show nutrient-dependent defenses under P-limitation.

One important goal for stoichiometric theory and TER models, in particular, is to predict when grazer growth will be P-limited in nature. The surprisingly few direct tests of Daphnia P-limitation in nature have provided mixed results. The best evidence for *Daphnia* P-limitation in nature comes from experimental testing in oligotrophic Canadian Shield lakes (Elser et al. 2001; Makino et al. 2002) and hypereutrophic Dutch lakes (DeMott et al. 2001). The importance of *Daphnia* P-limitation in the Dutch lakes gains further support from a 9-year study of between-year variation in seston C:P ratios and *Daphnia* abundance (DeMott and Gulati 1999; Van Donk et al. 2008). As predicted by TER models, high resource C:P ratios were associated with *Daphnia* P-limitation and low *Daphnia* abundance in both the Canadian and Dutch lakes. Several studies, however, provide no evidence for *Daphnia* P-limitation despite resources at or above predicted threshold C:P ratios, including research in temperate (DeMott and Tessier 2002; DeMott et al. 2004) and tropical (Fileto et al. 2007) lakes and polar ponds (Van Geest et al. 2007).

DeMott and Tessier (2002) used growth assays and isotope assimilation experiments to show that energy limitation due to digestion defenses accounted for the strong food quality limitation of *Daphnia* in deep, stratified Michigan lakes, despite resource C:P ratios well above nominal TER values. In contrast with shallow Michigan lakes and the Dutch and Canadian lakes mentioned above, the deep Michigan lakes were inhabited by high densities of large-bodied *Daphnia* and contained high relative abundance of algae with constitutive digestion defenses (Tessier and Woodruff 2002). Similar gradients of constitutive digestion defenses have been shown when grazer abundance was manipulated in field mesocosms (e.g., Porter 1973; Vanni 1997; Kerfoot et al. 1988). We (DeMott and Tessier 2002) showed that the interaction between algal defenses and algal P-limitation can lead to energy limitation in *Daphnia,* but we did not consider that digestion defenses could be strengthened when algal growth is nutrient limited.

Here, we use a laboratory microcosm experiment to test the effectiveness of algal defenses and their potential interactions with stoichiometric constraints. We manipulated grazers (*Daphnia* present or absent) and algal composition (single species cultures and mixtures of a defended and an undefended green alga). Our defended alga was a strain of *Oocystis* with a gelatinous sheath and thick cell walls that provide moderate constitutive defenses against digestion (see below). Using an alga with moderate constitutive defenses allowed us to test whether defenses would be enhanced under P-limited growth or induced by *Daphnia*’s presence. As in the studies by Elser et al. (2001), DeMott et al. (2001), and DeMott and Tessier (2002), we used juvenile growth assays with phosphate supplements to test for *Daphnia* P-limitation. Comparison of juvenile growth assays with resources from the grazed and control (*Daphnia*-free) microcosms tested for grazer-induced defenses.

Microcosm experiments can provide realistic feedbacks between grazer density, resource abundance, and resource C:P ratios. Previous microcosm experiments with strong P-limitation of algal growth provided evidence for density-dependent facilitation in *Daphnia* (Sommer 1992; Nelson et al. 2001; Urabe et al. 2002). In this situation, food conditions improve when *Daphnia* populations reduce the abundance of poorly defended, P-limited algae, causing a decline in the resource C:P ratio and a switch from *Daphnia* P-limitation to energy limitation. Comparison of the dynamics of the two algal species in the mixed treatment should provide the clearest basis for assessing the effectiveness of algal defenses. According to the algal defenses hypothesis (DeMott and Tessier 2002), effective defenses could lead to the coexistence of energy-limited *Daphnia* with high abundance of P-deficient, well-defended *Oocystis*.

## Materials and methods

### Experimental design

The effectiveness of algal defenses and their interactions with stoichiometric constraints were tested in a microcosm experiment that manipulated *Daphnia* presence and algal composition (undefended *Ankistrodesmus* and digestion-resistant *Oocystis* in single species cultures or a mixture). The microcosms were randomly positioned in 3 replicate blocks of 6 (2 grazer × 3 algae treatments; 18 total). The experiment was run for 25-days under nutrient and light conditions that were expected to produce strong P limitation of algal growth. Transparent Nalgene (Rochester, New York, USA) polycarbonate carboys were filled with 10-L of Combo medium (Kilham et al. 1998) with 10 μg P-phosphate L^−1^ and an N:P ratio of 40:1. The experiment was run in a temperature-controlled room (20 °C) with fluorescent lights (16 h light: 8 h dark) positioned about 1.2 m above the water level (PAR intensity measured at water level with a Li Cor model LI-189 photometer; mean ± SD = 96 ± 7.5 μmol s^−1^ m^−2^). Each microcosm was mixed by gentle continuous aeration and was lifted and swirled daily to resuspend particles that had settled to the bottom.

The grazer was a clone of a *Daphnia pulex* × *pulicaria* hybrid (Tessier and Woodruff 2002). This clone was the most sensitive to P-limitation of 10 *Daphnia* taxa tested by DeMott and Pape (2004). Animals for stocking the 9 *Daphnia* microcosms were fed P-rich *Ankistrodesmus* in excess, were grown in 5 cohorts comprising animals born within a 24-h period (range 0–12 days old), and were counted out individually (63 animals including 13 adults per microcosm).

The digestion-resistant *Oocystis*, which typically grew in colonies of 1–4 cells, was isolated from the guts of field-collected zooplankton about 8 months before the start of the experiment (mesotrophic Crooked Lake, Whitley and Noble counties, Indiana, USA). The digestion defenses of this gelatinous green alga were confirmed by visual observations on viable gut passage (DeMott et al. 2010) and by a preliminary juvenile growth assay that gave a growth rate of 0.37 ± 0.02 day^−1^ with a high food concentration (1 mg C L^−1^) from a P-sufficient culture. Previous studies with the same *Daphnia* clone and similar conditions gave juvenile growth rates ranging from 0.10 to 0.46 day^−1^ for 4 digestion-resistant green algae and from 0.57 to 0.61 for 3 undefended green algae, including *Ankistrodesmus* (DeMott et al. 2010). The undefended green alga, *Ankistrodesmus falcatus,* which grows in single, needle-shaped cells, was also used for culturing *Daphnia*. Estimates of *Daphnia* AE using ^32^P-labeled cells revealed a small but statistically significant decrease in the digestibility *Ankistrodesmus* with strong P-limitation (Ferrao-Filho et al. 2007). Algae from batch cultures growing in P-rich WC medium (Guillard and Lorenzen 1972) were centrifuged, resuspended in P-free Combo medium, and added to the microcosms at a concentration of 0.2 mg C L^−1^. The mixed treatment microcosms received 0.1 mg C L^−1^ of each algal species.

### Sampling, sample processing and data analysis

The initial (day 0) *Daphnia* population density was based on exact counts of animals stocked into each microcosm, while the initial estimates of particulate organic carbon (POC), particulate phosphorus (PP), and the resource C:P ratio were calculated from samples of the algal cultures added to the microcosms. Initial samples for estimates of particle volume and algal cell densities were collected from the microcosms with 25-mL glass pipettes.

Subsequent routine sampling used plexiglass tubes with an inner diameter of 56 mm and a volume of 400 mL (De Senerpont Domis et al. 2007). Before sampling, each microcosm was stirred with the sampling device to insure complete mixing. To reduce the chance of contamination, a separate sampling tube was used for each microcosm. Two tubes of water (800 mL) were collected from each microcosm on five sampling dates over 25 days, and the water was filtered through a 60-μm screen to collect *Daphnia*, which were preserved in a sucrose–formalin mixture. Sampling imposed a mortality rate of about 0.02 day^−1^ on the *Daphnia* and algal populations. The filtrate was used to estimate resource abundance (POC, PP, particulate volume, algal cell counts), the resource C:P ratio, and as a medium for juvenile growth assays. After sampling, Combo medium with phosphate was added to replace the volume sampled. Greater volumes were sampled for *Daphnia* on the penultimate (1.6 L) and final (3.2 L) sampling dates from microcosms with low grazer abundance.

All *Daphnia* in each sample were counted and measured using a dissecting microscope and Autoshape software (v.3.0; Comef). We determined clutch size as the number of parthenogenic eggs per adult female. The length of the smallest egg-bearing female was taken as the minimum size of the adult stage (1.50 mm).

POC, PP, and the C:P ratio were estimated from matter collected on the same glassfiber filter (GF/F, 25 mm diameter; Whatman, Maidstone, UK). Four small circles cut from each filter with a paper punch were analyzed for carbon as CO_2_ in a CN analyzer (Flash 2000; Interscience). The remaining filter was ashed for 30 min at 550 °C, dissolved in 10 mL of 2 % persulfate, and analyzed as orthophosphate according to Murphy and Riley (1962), using an autoanalyser (Quaatro; Seal Analytical, Beun de Ronde, Abcoude, The Netherlands). A number of spuriously low PP values (6 of 90) were replaced with the mean of the previous and following sampling dates.

The volume of algae, detritus, and bacteria (0–30 μm diameter) was estimated using a CASY model TTC (Shärfe System) electronic particle counter with a 100-μm aperture. Samples were collected and analyzed daily during the first 4 days and every second day thereafter. On days when tube samples were not collected, samples were collected with 25-mL glass pipettes. The particle counter apparently sensed *Oocystis* cells individually, and thus did not provide useful data on *Oocystis* colony size.

Algal densities were estimated from samples preserved in acidified Lugol’s solution and counted at ×200 using an inverted microscope. Estimates for each sample and species were based on counts of at least 100 colonies or cells and 8 fields of view. The effect of *Daphnia* presence on *Oocystis* size was tested by measuring the diameter of *Oocystis* colonies from the 12 microcosms in the *Oocystis* and mixed treatments on days 3 and 20. Photographs were taken at ×400 of 6–12 random fields from each sample. Diameter (mean of length and width of ovoid colonies) was estimated for each colony in clear focus (range 37–95 colonies per sample) using Cell D Software (v.1.2; Olympus).

### *Daphnia* juvenile growth assays

Since P-deficient algae take up phosphate from solution within minutes, short-term growth assays with phosphate supplements provide direct tests of *Daphnia* P-limitation. P supplements typically restore much but not all of the reduced growth of *Daphnia* fed undefended algae cultured under P-deficient conditions (DeMott 1998; Boersma 2000; Plath and Boersma 2001). Thus, we tested the intensity and mechanism of food limitation by growing newborn *Daphnia* for 4 days in resources from the microcosms with and without phosphate supplements (0.5 μmol L^−1^). All growth assays were run using cohorts of *Daphnia* born within the previous 24 h to mothers fed high concentrations of P-sufficient *Ankistrodesmus*, and were run in the temperature-controlled room (20 °C) and in the dark to prevent algal growth and to limit potential changes in algal biochemistry or morphology. Since *Daphnia* feeding on P-deficient resources show declines in body P-content and produce young with reduced P-content (DeMott et al. 1998), single generation growth essays underestimate stoichiometric constraints on *Daphnia* growth (Frost et al. 2010).

Growth assays started on days 6, 11, 15, and 20 were run with resources from the three *Daphnia* treatments and the ungrazed *Oocystis* treatment. Thus, on each of the four dates, we started an experiment with 8 treatments (2 phosphorus × 4 algal treatments). In addition, on each of these 4 days, and on day 0, we ran treatments with *Ankistrodesmus* and *Oocystis* from cultures growing exponentially in P-rich WC medium at a concentration of 1.0 mg C L^−1^. These positive controls tested whether animals would show the expected high growth rates with P-rich resources. The growth assays were run with 3 *Daphnia* in 100 mL of medium and the medium was changed after 2 days. On days when the medium was not changed (days 1 and 3), particles that had settled to the bottom were resuspended with a glass pipette. Water collected from each microcosm was stored in a beaker in the dark for use in one replicate of a growth assay and the corresponding P-enriched beaker. Thus, the bioassays reflected resource variation between the replicate microcosms.

Lipid reserves can provide clues to whether *Daphnia* growth is limited by P or energy. Animals experiencing P-limitation assimilate carbon in excess of energy requirements and may accumulate substantial lipid reserves despite low growth (Sterner et al. 1992). In contrast, energy limitation and starvation lead to low lipid reserves (Tessier et al. 1983). At the end of each growth assay, a visual lipid–ovary index was estimated for each individual on a scale from 0 (no lipid reserves) to 3 (maximal lipids; Tessier et al. 1983). The animals were then dried and weighed to the nearest μg. The growth rate (g day^−1^) was calculated as the difference between the natural logarithm of the final and the initial dry mass, divided by the experiment duration (4 days). P-differentials were calculated by subtracting the growth rate for a control beaker from growth for the beaker with added phosphate from the same microcosm (DeMott and Tessier 2002).

A final series of growth assay experiments was designed to test mechanisms for the poor quality of P-deficient resources. Phosphate additions should reduce the algal C:P ratio below potentially limiting levels within minutes. However, we hypothesized that phosphate, light, and sufficient time for cell division would be needed to overcome the nutrient-dependent digestion defenses of thickened cell walls and gelatinous sheaths. Preparation for this experiment was started on day 25 using composite samples from the strongly P-deficient, ungrazed *Ankistrodesmus* and *Oocystis* microcosms. Each algal species was grown in 3 flasks under 3 sets of conditions for 3 days. In the P-deficient treatment, 1 L of algae was kept in the light without added phosphate. In second and third treatments, 800 mL of microcosm resources were diluted with 200 mL of P-rich WC algal medium and incubated in the light or in the dark, respectively. The growth assays included five treatments for each algal species: (1) P-sufficient laboratory culture control, (2) added WC medium incubated in light, (3) added WC medium incubated in the dark, (4) P-deficient treatment +0.5 μmol phosphate L^−1^, and (5) P-deficient treatment. These growth assays followed the same methods as the previous experiments except that the medium was changed daily and the food concentration was diluted to 1 mg C L^−1^. Limiting the food concentration insured that the algal C:P ratio would be reduced below nominal TER values (<200) in treatments with added phosphate and WC medium. The phosphate supplement in treatment 4 was added immediately after the daily medium change. Samples were filtered from the 6 stock culture flasks to estimate the resource C:P ratios.

### Statistical analysis

The effects of algal treatment on *Daphnia* dynamics were analyzed by one-way ANOVA while the effects of grazers on their resources were analyzed by two-way ANOVA (grazer × algal species). A *Daphnia* × algae species interaction provides evidence of differences between *Ankistrodesmus* and *Oocystis* in their susceptibility to *Daphnia* grazing. Pairwise comparisons were based on the Holm–Sidak method. The parameter value from each microcosm was the mean from day 6 (when the first zooplankton samples were taken) until the end of the experiment (day 25). For juvenile growth assays, each P-differential was testing using the *t* distribution to determine whether the 95 % confidence interval overlapped with zero. With the exceptions of exponential rates of individual and population growth, data were transformed to equalize variances (log transformation of measurements; arcsin transformation of proportions).

## Results

### *Daphnia* dynamics

Algal species composition strongly affected *Daphnia* population dynamics, including population density, clutch size, and the proportion of adult females (three one-way ANOVAs, *F* = 13.2–128, each *p* < 0.01). *Daphnia* dynamics reflected similarities between the *Ankistrodesmus* and mixed treatments (3 pairwise contrasts, each *p* > 0.05) and strong divergences between the *Oocystis* treatment and the other two treatments (6 contrasts, each *p* < 0.01). As described below, *Daphnia* population growth, reproduction, and survival were much lower in the *Oocystis* treatment.

**Fig. 1:**
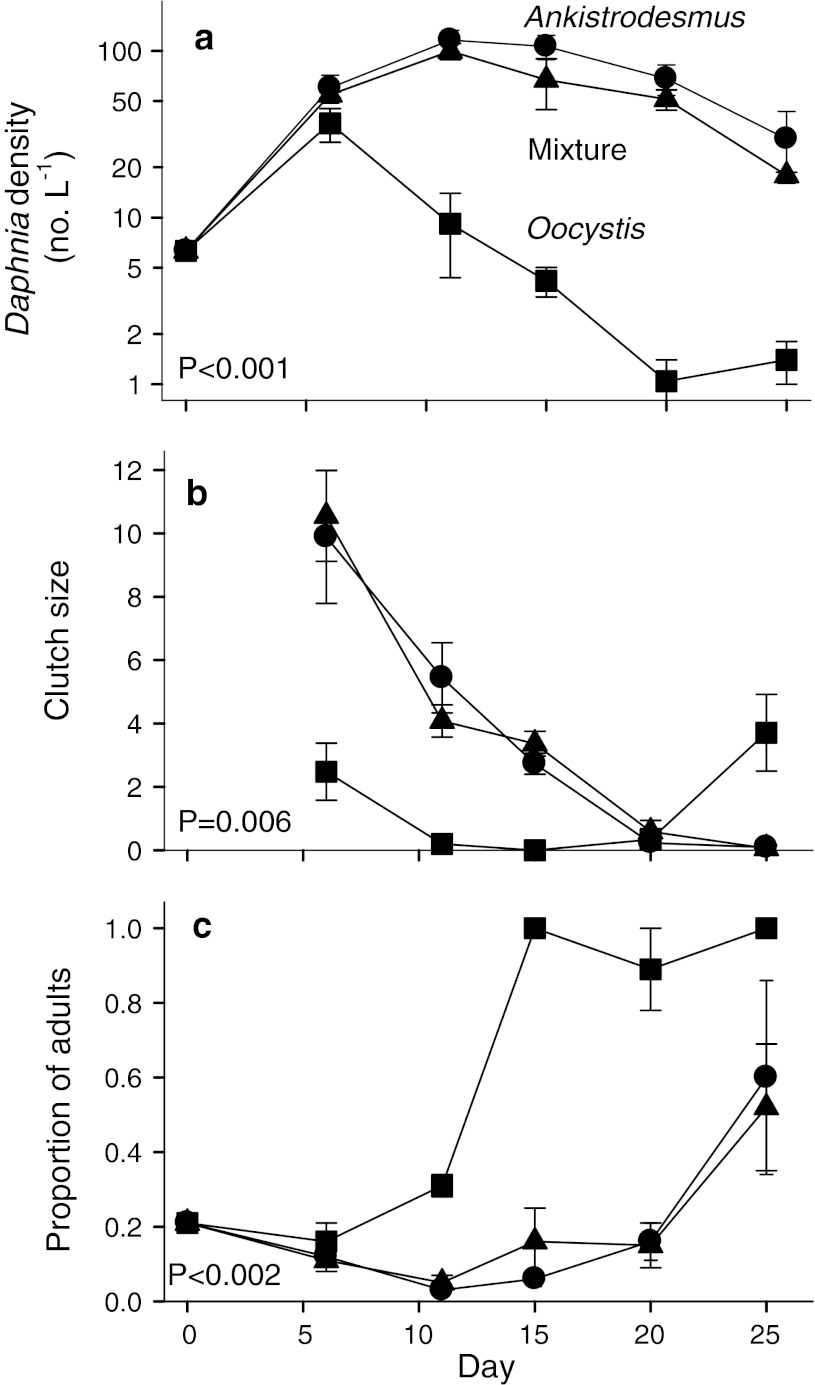
Effects of three algal treatments on **a** *Daphnia* population density, **b** clutch size, and **c** proportion of adults during the 25 day experiment. Algal treatments include *Ankistrodesmus* (*circles*), *Oocystis* (*squares*), or a mixture (*triangles*). Data are mean ± SE for 3 replicate mesocosms. *p* values are from one-way ANOVAs. Note log scale in (**a**)

*Daphnia* populations increased in all three treatments during the first 6 days of the experiment (Fig. 1a). However, from day 6 to day 20, *Daphnia* in the *Oocystis* treatment declined sharply, while populations in both the *Ankistrodesmus* and mixed treatments showed gradual increases and slow declines over the same period.

The divergences in *Daphnia* abundance between the *Oocystis* treatment and the other two treatments were corroborated by data on reproduction. Clutch size in the *Oocystis* treatment declined from 2.5 ± 0.9 on day 6 to near-zero values from days 11–20 (Fig. 1b). Clutch size in the other two treatments declined from high, nearly identical levels on day 6 to near-zero values on days 20 and 25.

Declines in the proportion of adults early in the experiment can be attributed to reproduction and poor conditions for juvenile growth (Fig. 1c). However, between days 11 and 15, the proportion of adults in the *Oocystis* treatment increased from 0.31 ± 0.028 to 1.00, as body length increased from 1.09 ± 0.06 to 1.90 ± 0.06 mm. The rapid increases in both body size and the proportion of adults during this period of near-zero reproduction (Fig. 1b) and population decline (Fig. 1a) were probably due to nearly complete juvenile mortality, rather than rapid juvenile growth.

### Resource dynamics

Particle volume (0–30 μm) was closely correlated with POC measured from the same sample (log–log plot; *r* ^2^ = 0.97; data not shown). Both algal species showed maximal exponential growth in biovolume from days 1 to 3 in their respective controls (log particle volume versus time; *Ankistrodesmus*, *r* ^2^ > 0.99; *Oocystis*, *r* ^2^ = 0.92–0.98). The maximal exponential growth rate of *Ankistrodesmus* (0.93 ± 0.01 day^−1^) was greater than that of *Oocystis* (0.63 ± 0.01 day^−1^; *t* = 16.8, *p* < 0.001).

**Fig. 2:**
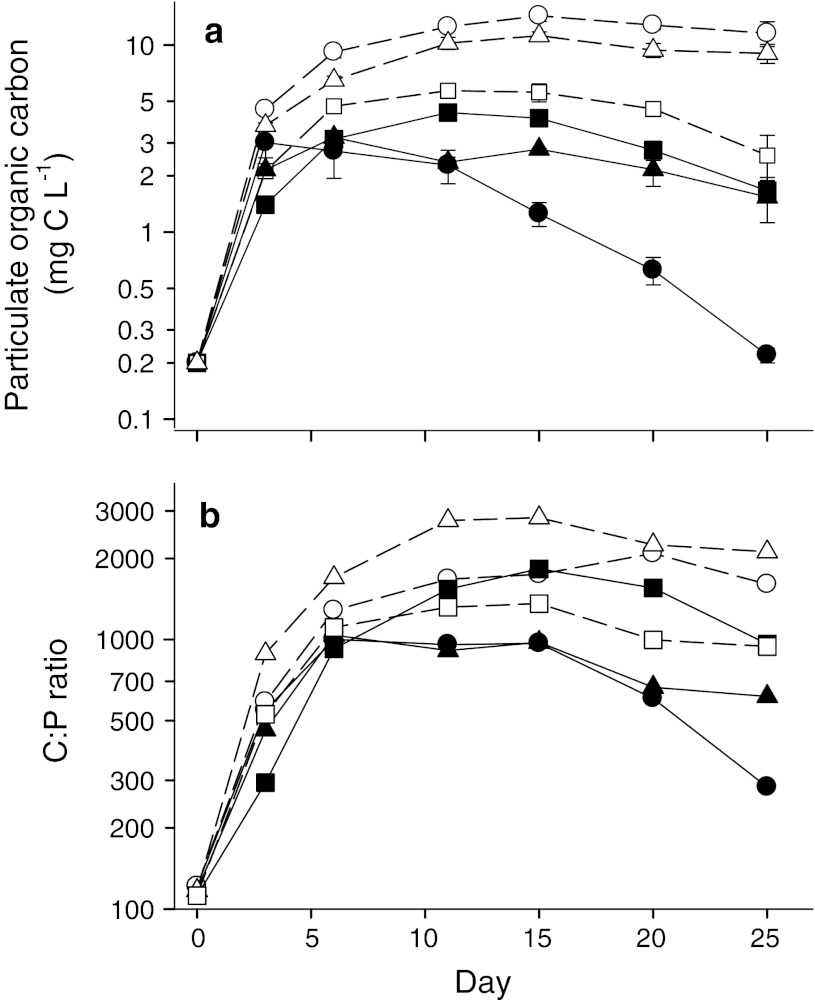
Effects of *Daphnia* presence (*filled symbols*) or absence (*open symbols*) and three algal treatments on **a** particulate organic carbon, and **b** molar C:P ratio. Algal treatments include *Ankistrodesmus* (*circles*), *Oocystis* (*squares*), or a mixture (*triangles*). Data are mean ± SE for replicate microcosms. *Error bars* were removed from (**b**) for clarity. Note log scales

Particulate organic carbon (POC) was reduced in the *Daphnia* treatment (*F* = 484, *p* < 0.001) and the *Daphnia* effect differed between algal treatments (*Daphnia* × algae interaction: *F* = 78; *p* < 0.001; Fig. 2a). Moreover, POC was lower in the *Ankistrodesmus* treatment than the other two treatments (2 contrasts, each *p* < 0.02). Resources remained abundant in the mixed and *Oocystis* treatments. Thus, the sharp decline in *Daphnia* abundance in the *Oocystis* treatment occurred despite consistently high food abundance (range 1.4–4.7 mg C L^−1^ from day 6 to final day).

All treatments showed sharp increases in the C:P ratio over the first 6 days to values far above the nominal TER for *Daphnia* P-limitation (Fig. 2b). On day 6, the mean C:P ratio ranged from 910 to 1,000 in the *Daphnia* treatments and from 1,110 to 1,700 in the ungrazed controls. From day 6 to the end of the experiment, the resource C:P ratio was lower in the *Daphnia* treatment (*F* = 64.5, *p* < 0.001) and the *Daphnia* effect differed between algae treatments (*Daphnia* × algae interaction; *F* = 25.0, *p* < 0.001). Pairwise comparisons show significant reductions of the C:P ratio in the *Daphnia* treatment for the *Ankistrodesmus* and mixed treatments relative to their respective controls (both *p* < 0.001), but not in the *Oocystis* treatment (*p* = 0.33). With the exception of the last sampling date in the *Ankistrodesmus* treatment (mean 284 ± 26), the C:P ratio remained well above the nominal threshold for *Daphnia* P-limitation in all treatments from day 6 until the end of the experiment (C:P >600; Fig. 2b).

**Fig. 3:**
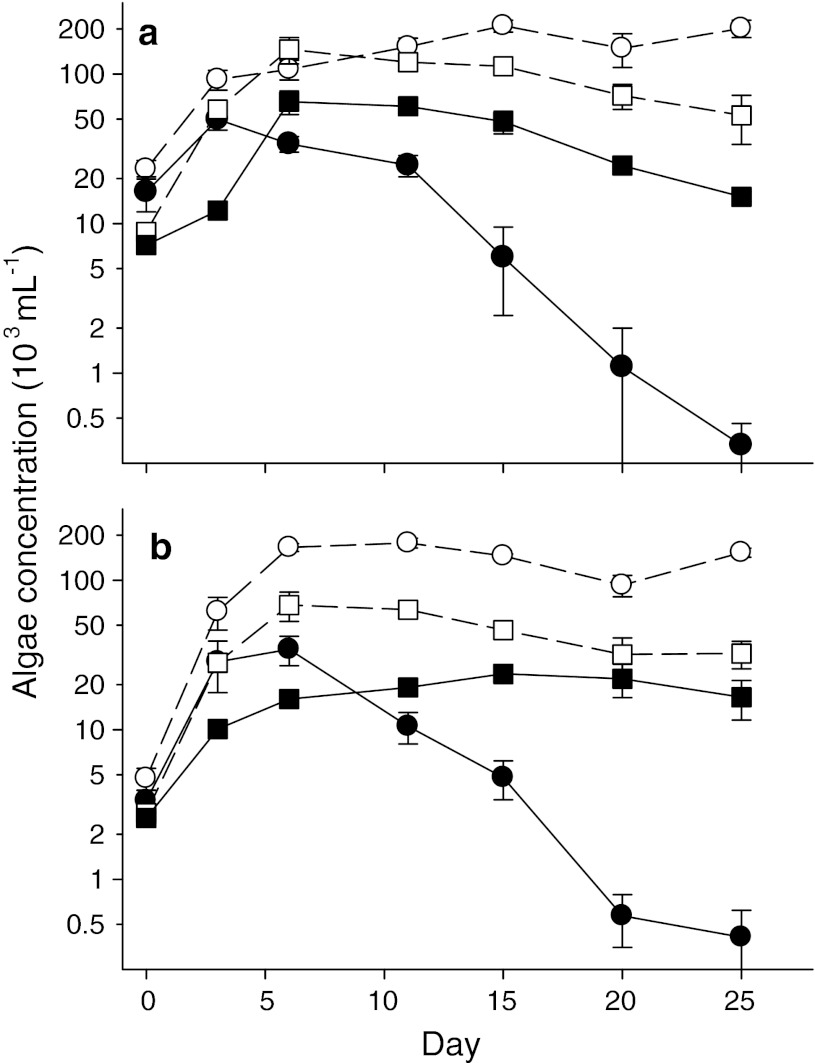
Effects of *Daphnia* presence (*filled symbols*) or absence (*open symbols*) on the population dynamics of *Ankistrodesmus* (*circles*) and *Oocystis* (*squares*) in the **a** single algal species and **b** mixed species treatments. Data are mean ± SE for 3 replicate microcosms. Note log scales

Consistent with the POC data, algal cell counts demonstrate very strong differences between *Ankistrodesmus* and *Oocystis* in their vulnerability to suppression by *Daphnia* grazing (Fig. 3). Separate two-way ANOVAs showed strong *Daphnia* effects and *Daphnia* × algae interactions in both the single species (Fig. 3a; *Daphnia* effect, *F* = 247; interaction, *F* = 66.1; both, *p* < 0.001) and mixed treatments (Fig. 3b; *Daphnia* effect, *F* = 128; interaction, *F* = 34.1; both, *p* < 0.001). In both the single species and mixed treatments, *Ankistrodesmus* showed the highest cell counts in the control but the lowest cell counts in the *Daphnia* treatment (Fig. 3). By the last sampling date, *Ankistrodesmus* had declined in the *Daphnia* treatment to values <1 % of its controls in both the single species and mixed treatments, whereas *Oocystis* in the *Daphnia* treatments were 29–51 % of control values on the same date (Fig. 3). On the last sampling date, *Ankistrodesmus* comprised 82.5 ± 3.5 % of the algae in the mixed ungrazed treatment, but only 2.5 ± 0.9 % of the algae in the mixed *Daphnia* treatment.

We directly compared the performance of the two algal species by calculating mean population growth rates (*r*, day^−1^) over the interval from day 6 to the end of the experiment. Over the final 19 days of the experiment, *Ankistrodesmus* declined sharply in the mixed algae *Daphnia* treatment (*r* = −0.24 ± 0.032 day^−1^) while *Oocystis* remained abundant and stable in the same microcosms (*r* = −0.005 ± 0.016 day^−1^; *p* < 0.001). In contrast, both populations of both species were relatively stable in the controls, although *Ankistrodesmus* had a higher growth rate than *Oocystis*, and this difference was significant in the single species treatment (*p* < 0.02).

Measurements of *Oocystis* diameter on days 3 and 20 show that the presence of *Daphnia* favored larger colony size, and this effect increased over time (two-way ANOVA: *Daphnia* effect, *F* = 74.1, *p* < 0.001; *Daphnia* × day interaction, *F* = 12.9, *p* = 0.002). The magnitude of this grazer effect was, however, small (mean diameters of 12.0 ± 0.25 and 15.2 ± 0.31 μm in the control and *Daphnia* treatments, respectively, on day 20).

### *Daphnia* juvenile growth assays

**Fig. 4:**
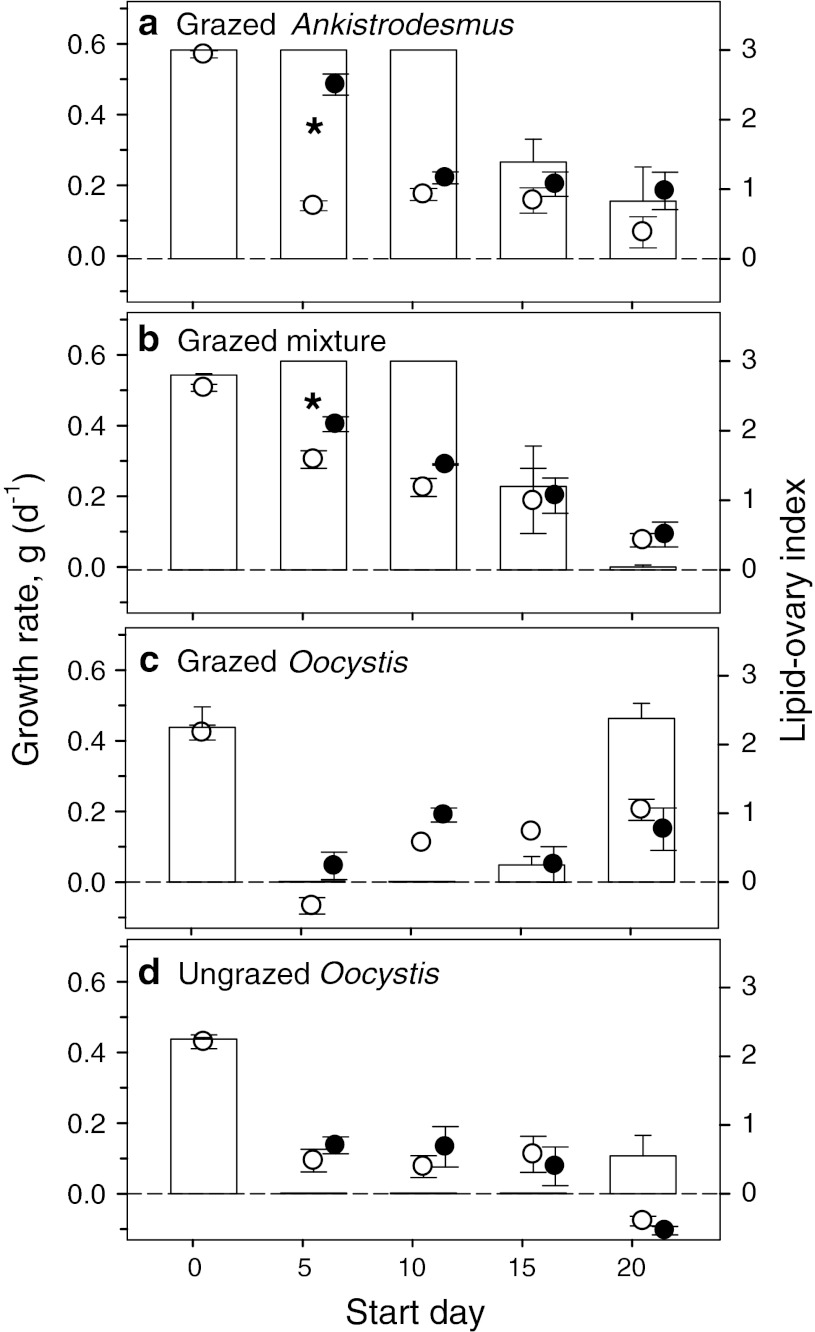
The effects of mesocosm resources and phosphate supplements on *Daphnia* juvenile growth (*circles*) and lipid-ovary index (*bars*). The day 0 trials used P-sufficient laboratory cultures (laboratory culture). Subsequent experiments used mesocosm resources from the **a** grazed *Ankistrodesmus*, **b** grazed mixture, **c** grazed *Oocystis* and **d** ungrazed *Oocystis* microcosms. Responses to phosphate supplements (*solid circles*) relative to controls (*open circles*) are shown by *asterisks* (*) for *p* < 0.05. Growth data are mean ± SE for resources from 3 replicate beakers. The lipid ovary index was scored on a scale of 0–3 and shows overall mean ± SE for both the control and supplemented trials. The value of the lipid-ovary index is zero for experiments starting on days 6 and 11 in (**c**) and days 6, 11, and 15 in (**d**)

Juvenile growth assays provide information on the intensity and mechanism of *Daphnia* food limitation that supports and complements our analysis of the microcosm populations. An initial experiment with algae from the P-sufficient cultures used to inoculate the microcosms confirmed that juvenile growth is very high with *Ankistrodesmus*, intermediate with the algal mixture, and lowest but still moderately high with *Oocystis* (day 0; Fig. 4). Experiments with P-sufficient laboratory cultures starting on days 6, 11, 15, and 20 showed similar high growth rates for *Ankistrodesmus* (range 0.57–0.61 day^−1^) and moderately high rates for *Oocystis* (0.42–0.52 day^−1^; data not shown).

Weight loss (i.e., negative growth) in the control grazed *Oocystis* bioassay begun on day 6 (Fig. 4c) corresponds with the beginning of the sharp decline in *Daphnia* abundance in the *Oocystis* microcosms, while substantially higher growth in the *Ankistrodesmus* and mixed control bioassays (Fig. 4a, b) corresponds to the period of slow *Daphnia* increase in the corresponding microcosms (Fig. 1a). Since algae were abundant in the microcosms in all three algal treatments from days 6–11 (Fig. 2a; POC range 1.2–4.7 C L^−1^), sharply reduced growth in both the microcosms and the growth assays can be attributed to reduced food quality, not low quantity.

In four growth assay experiments (excluding day 0), the overall mean *Daphnia* control growth rate was 0.10 ± 0.06 day^−1^ for resources from the grazed *Oocystis* microcosms and 0.05 ± 0.04 day^−1^ for the ungrazed (control) microcosms (Fig. 4c, d). Thus, there is no evidence that the strong defenses of P-deficient *Oocystis* were induced by *Daphnia*’s presence.

Responses to phosphate supplements suggest that P-limitation reduced *Daphnia* growth in both *Ankistrodesmus* and mixed treatments in the growth bioassays begun on day 6 (Fig. 4a, b). P-differentials were positive for the *Ankistrodesmus* and mixed treatments for the final 3 experiments, but were not statistically significant, even though the resource C:P ratio ranged from 980 to 600.

*Daphnia* showed no statistically significant responses to P supplements in any of the trials with resources from the grazed or ungrazed *Oocystis* microcosms (Fig. 4c, d). Moreover, the mean values for mesocosm resources were higher than the mean P-supplement values in 4 of the 8 comparisons. Resource C:P ratios remained above 900 in all of the *Oocystis* microcosms.

Based on POC and PP estimates from the microcosms, and assuming complete uptake of the phosphate supplements, the resource C:P ratios were reduced (ranges of means for experiments) from 285–1,000 (control) to 32–310 (phosphate supplement) for *Ankistrodesmus*; from 615–1,040 to 180–350 for the mixed treatment; from 960–1,830 to 215–490 for grazed *Oocystis* and from 940–1,360 to 290–545 for control *Oocystis*. Thus, due to the extreme algal P-limitation, the phosphate supplements strongly reduced but did not eliminate the potential for *Daphnia* P-limitation.

The lipid–ovary index provides further evidence of differences in the mechanism of food limitation between the *Oocystis* and the *Ankistrodesmus* and mixed treatments. For resources from both the grazed and ungrazed *Oocystis* microcosms, zero lipid values from experiments begun on days 6 and 11 suggest extreme energy limitation (Fig. 4c, d). In contrast, maximal lipid values for the *Ankistrodesmus* and mixed treatments on the same dates are consistent with P-limitation (Fig. 4a, b). The shift in the *Ankistrodesmus* assays from maximal to moderate lipid reserves between days 15 and 20 occurred as the food concentration decline from 1.3 to 0.6 mg C L^−1^ and the resource C:P ratio declined from 970 to 600.

**Fig. 5:**
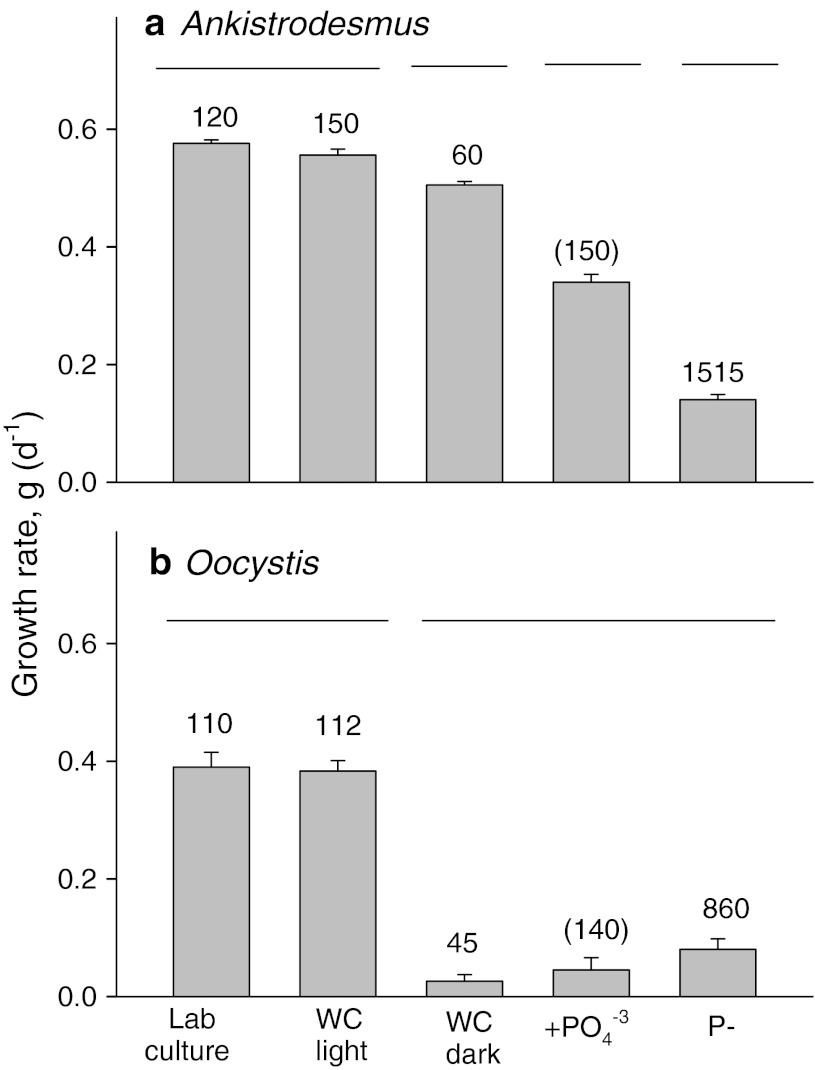
*Daphnia* juvenile growth assays with 1.0 mg C L^−1^ of algae from P-sufficient laboratory cultures (laboratory culture) and from the ungrazed microcosms of **a** *Ankistrodesmus* and **b** *Oocystis*. Resources from microcosms were incubated for 3 days in the light without nutrients or with WC algal medium in light (*WC light*) or dark (*WC dark*). The mesocosm resources without WC medium were supplemented with phosphate each day of the growth experiment (+PO_4_^−3^) or left untreated (P−). Treatments not connected by *lines* are significantly different (Holm–Sidak procedure, *p* < 0.05). The C:P ratio (shown above each *bar*) was calculated from the concentration of added phosphate (*numbers in parentheses*) or measured from 3 replicate filters

Growth assays using resources from the ungrazed *Ankistrodesmus* and *Oocystis* microcosms at the end of the experiment provide further evidence for differences in the mechanism of poor food quality for P-limited cultures of these two taxa. First, growing P-deficient cultures in the light for 3 days with P-rich WC algal medium fully restored food quality for both algal species (laboratory culture vs. WC light; Fig. 5). Comparisons between the two WC treatments (light vs. dark) support our hypothesis that phosphorus, light, and enough time for cell division are needed to reduce the nutrient-dependent defenses of *Oocystis* to the baseline constitutive level (Fig. 5b). In contrast to *Oocystis*, growing *Ankistrodesmus* with the P-rich WC medium in the dark for 3 days improved *Daphnia* growth to almost the same level as the laboratory culture control, supporting the P-limitation hypothesis (Fig. 5a). However, daily phosphate supplements in the dark (+PO_4_^−3^) only partially ameliorated the poor food quality of strongly P-deficient *Ankistrodesmus* (P-), suggesting that unknown factors may also play a role in poor food quality. As in the previous growth assays (Fig. 4), short-term supplementation of P-deficient *Oocystis* with phosphate did not improve *Daphnia* growth.

*Daphnia* feeding in all five *Ankistrodesmus* treatments were rich in lipids suggesting that energy limitation was weak or absent in each treatment (Table 1). In contrast, *Daphnia* feeding on *Oocystis* showed moderate lipid levels when feeding on cultures rapidly growth in the light (laboratory and WC + light) and low lipids when feeding on the P-deficient culture (P−) or P-enriched cultures kept in the dark (WC dark and +PO_4_^−3^). Thus, as in the previous series of growth assays (Fig. 4), *Daphnia* feeding on P-deficient *Oocystis* showed no evidence of P-limitation.

**Table 1:** Effects of treatments on lipid ovary index (scale 0–3) in growth assays with resources from P-sufficient laboratory cultures (*Lab*) and from the ungrazed *Ankistrodesmus* (*Ank*) and *Oocystis* microcosms

	Lab	WC light	WC dark	+PO_4_^−3^	P−	*F*	*p*
*Ank*	3.0 a	3.0 a	3.0 a	3.0 a	2.94 ± 0.06 a	1.00	0.45
*Oocystis*	2.28 ± 0.20 b	2.14 ± 0.28 b	0.90 ± 0.21 c	0.33 ± 0.04 c	1.08 ± 0.17 c	18.6	<0.001

Mesocosm resources were incubated for 3 days in the light without nutrients or with P-rich WC algal medium in the light (WC light) or dark (WC dark). The resources without WC medium were supplemented with phosphate each day of the growth experiment (+PO_4_^−3^) or left untreated (P−). Data are mean ± SE for three replicate beakers. Data with different letters within rows are significantly different (Holm-Sidak, *p* < 0.05)

We conducted 47 juvenile growth assays with P-rich laboratory cultures and P-deficient and phosphate supplemented resources from the *Ankistrodesmus* and *Oocystis* microcosms. Based on median values, *Ankistrodesmus* was a better resource than *Oocystis* with both P-rich laboratory cultures (0.58 vs. 0.40 day^−1^) and P-deficient (0.13 vs. 0.10 day^−1^) microcosm resources. However, the two resources differed qualitatively in grazer responses to phosphate supplements in the dark (medians: *Ankistrodesmus*, 0.29 day^−1^; *Oocystis*, 0.06 day^−1^). Direct P-limitation explains part of the poor quality of P-*Ankistrodesmus* (36 % of the difference between medians of P-rich laboratory cultures and P-deficient microcosms resources), while there is no evidence of P-limitation for juvenile *Daphnia* feeding on strongly P-deficient *Oocystis* (−13 % of the difference between P-rich laboratory cultures and P-deficient microcosm resources).

## Discussion

This study provides evidence that P-limitation enhances the defenses of a digestion-resistant alga, favoring high abundance of strongly defended algae and energy limitation of *Daphnia* growth. Although earlier studies showed nutrient-dependent digestion defenses in green algae that were undefended, high quality resources under nutrient-sufficient conditions (Van Donk and Hessen 1993; Van Donk et al. 1997), this study is the first to show that P-limitation enhances the defenses of an alga with constitutive digestion defenses. Algal dynamics provide a marked contrast between strong defenses in P-deficient *Oocystis* and weak, ineffective defenses in P-limited *Ankistrodesmus*. Thus, we found very strong differences between two species of green algae in the degree of defense enhancement under P-limitation.

*Daphnia* population dynamics in the microcosms indicate that food quality was much lower in the *Oocystis* treatment. Juvenile growth assays indicate that our strain of *Oocystis* is a good, although suboptimal, resource when grown under P-sufficient conditions (median growth assay 0.40 day^−1^), but a very poor quality food when P-deficient (0.10 day^−1^). Using the same *Daphnia* clone, DeMott et al. (2010) found that juvenile growth rate was tightly correlated with juvenile carbon assimilation efficiency (AE; *r* ^2^ = 0.96) among seven taxa of green algae that varied in digestion defenses. Viable gut passage in the digestion-resistant taxa implies that the cell wall is not breached and that neither C nor P is assimilated. Mean *Daphnia* juvenile growth rate on high concentrations of the P-sufficient algae ranged from 0.57 to 0.61 for three undefended taxa and from 0.10 to 0.46 day^−1^ for four digestion-resistant taxa, including two strains of *Oocystis*. Thus, the strain of *Oocystis* used in the microcosms, when grown under a range of nutrient conditions, spans nearly the full range in food quality of the four digestion-resistant algae studied by DeMott et al. (2010).

Using the AE versus growth relationship from DeMott et al. (2010), juvenile *Daphnia* feeding on the P-sufficient *Oocystis* in the growth assays are predicted to assimilate carbon with 41 % efficiency, while the same strain under P-deficient conditions would be assimilated with only 7 % efficiency. Clearly, such a low AE is consistent with a high frequency of viable gut passage and a strongly enhanced digestion defense. Thus, application of TER models to algae with strong, nutrient-dependent digestion defenses may require C and P assimilation efficiencies <0.1.

Although we did not directly verify energy limitation in *Daphnia* feeding on *Oocystis*, our results are contrary to three important alternatives to the nutrient-dependent digestion-resistance hypothesis. First and most important, juvenile growth assays with phosphate supplements provide no evidence for even weak P-limitation of *Daphnia* feeding on strongly P-deficient *Oocystis* from the microcosms. Since phosphate supplements can saturate C:P ratios of P-deficient algae within minutes, growth assays with phosphate supplements are the “gold standard” for demonstrating zooplankton P-limitation (DeMott 1998; Plath and Boersma 2001). We hypothesized that light, added phosphate, and sufficient time for cell division would be needed to reduce the nutrient-dependent digestion defenses of P-deficient *Oocystis* back to the baseline constitutive level, and this hypothesis was supported in our final series of growth assays. The lack of evidence for P-limitation in *Daphnia* feeding on *Oocystis* is strengthened by the positive growth responses for *Daphnia* feeding on P-deficient *Ankistrodesmus* with phosphate supplements. The very low lipid reserves of *Daphnia* feeding on P-deficient *Oocystis* are also consistent with strong energy limitation and contrast with the high lipid reserves of P-limited *Daphnia* feeding on high concentrations of P-deficient *Ankistrodesmus*. The phosphate supplements substantially reduced resource C:P ratios, in some, but not all cases, to below nominal TER values. On the other hand, while population dynamics in the microcosms included transgenerational effects of P-deficient resources (Frost et al. 2010), most growth assays, including ours, did not.

Since P-deficient *Oocystis* from both the grazed and control microcosms were very poor resources, we conclude that algal P-limitation, rather than a grazer-induced defense, was responsible for the extremely poor quality of the P-deficient algae in the grazed *Oocystis* microcosms. We cannot rule out the possibility, however, that *Oocystis* might show an induced digestion defense when grown in the presence of grazers under P-sufficient conditions. Kampe et al. (2007) showed that the presence of *Daphnia* caused a shift toward large, inedible colonies in the gelatinous green alga *Sphaerocystis.* We also found that *Daphnia* caused an increase in *Oocystis* colony size. However, the increase from 12 to 15 μm in diameter for *Oocystis* compares to an increase from 8–20 to 30–200 μm observed in *Sphaerocystis*. Based on a gut content study, DeMott (1995) found that small (1 mm) *Daphnia* readily ingested a spherical gelatinous green alga (*Eurdorina*) up to 40 μm in diameter, while large (2 mm) *Daphnia* showed an upper limit of about 80 μm. Thus, for *Daphnia* feeding on P-deficient *Oocystis*, our results are contrary to *Daphnia* P-limitation, while we show that *Oocystis* from both the grazed and ungrazed mesocosms were readily ingested even by small *Daphnia*. Although the P-deficient *Oocystis* was clearly a poor resource for both adults and juveniles, the evidence for nearly complete juvenile mortality in the *Oocystis* treatment is consistent with the juvenile bottleneck hypothesis (DeMott et al. 2010).

Laboratory studies testing for *Daphnia* P-limitation typically have used P-sufficient and P-deficient cultures of a single, readily ingested and digested, undefended green alga. This approach controls for taxonomic variation while maximizing the chance of finding evidence for zooplankton P-limitation. However, as our study shows, the results may be misleading, because natural algal assemblages include many species that vary in maximal growth rate, competitive ability, and defenses against grazers. While there are no comparable laboratory studies, our results are very similar to those of DeMott and Tessier (2002), who studied deep, stratified Michigan lakes with high densities of large-bodied *Daphnia* that coexisted with high abundance of P-limited digestion-resistant algae during summer (Tessier and Woodruff 2002).

Throughout the microcosm experiment, *Daphnia* abundance and reproduction were very similar in the mixed and the *Ankistrodesmus* treatments. This suggests that *Daphnia* mainly responded to the abundance of *Ankistrodesmus* and that *Oocystis* in the mixed treatment had very little positive or negative, interfering effect. Notably, *Daphnia* in both the mixed and *Ankistrodesmus* treatments showed evidence of similar, very strong energy limitation by the end of the experiment, when *Ankistrodesmus* had declined to low densities in the grazed *Ankistrodesmus* and the grazed mixed treatment mesocosms. Thus, in all three grazed treatments, the mesocosms approached equilibria with strongly energy-limited *Daphnia*. Ironically, by the end of the experiment, algal P-limitation was strongest in the *Oocystis* and mixed treatments where it acted to enhance *Oocystis’* defenses. The rapid declines of *Ankistrodesmus* in the single species and mixed treatments clearly depended on high *Daphnia* density and strong grazing.

With only a few exceptions, stoichiometric models have assumed homogeneous, undefended resources (reviewed by Andersen et al. 2004; Moe et al. 2005). One effect of including inedible (Hall et al. 2006) or digestion-resistant (Hall et al. 2007) algae in stoichiometrically explicit food chains is to elevate and prolong algal nutrient limitation. This occurs because inedible and well-defended algae remain abundant despite intense grazing. Supporting these predictions, algal densities and resource C:P ratios in the *Daphnia* microcosms were highest in the *Oocystis* treatment and lowest in the *Ankistrodesmus* treatment. The theoretical models also predict that predation on grazers increases grazer and algal nutrient limitation by reducing grazing intensity (Hall et al. 2007). Since Hall et al. (2007) assumed that digestion resistance is a fixed trait, our finding that digestion resistance is enhanced by nutrient limitation adds a new dimension for theoretical analysis and empirical study.

In agreement with other empirical studies (reviewed by Agrawal 1998), our results suggest a tradeoff between algal defenses and maximal growth rate that is consistent with theory on how algal defenses help account for the dynamic outcome of interactions between multi-species assemblages of phytoplankton and their grazers (Grover 1995; Grover and Holt 1998). Stronger evidence for an evolutionary tradeoff would require comparisons between closely related taxa, such as different strains of *Oocystis* (Yoshida et al. 2004). Under our experimental conditions, maximal growth rate was only a factor during the first few days, before the onset of severe nutrient limitation. However, phytoplankton can experience prolonged periods of near-maximal cell division when intense grazing keeps populations at low levels. Such conditions may favor fast-growing undefended algae over slower-growing, well-defended species (Grover and Holt 1998; Sarnelle 2005). Low food abundance and larger grazer body size also reduce the effectiveness of digestion defenses by slowing gut passage (DeMott et al. 2010). Thus, understanding the costs and benefits of defense is very important for predicting when well-defended algae will dominate phytoplankton assemblages. Our results show that stoichiometric theory needs to be better integrated with theory on algal defenses. Such a theory will need to consider stoichiometric constraints together with the costs of defense, the enhancement of digestion defenses with nutrient limitation, and the effects of food concentration and grazer body size in modulating the effectiveness of digestion defenses.
